# Adult Patients with Respiratory Distress: Current Evidence-based Recommendations for Prehospital Care

**DOI:** 10.5811/westjem.2020.2.43896

**Published:** 2020-06-25

**Authors:** Sammy S. Hodroge, Melody Glenn, Amelia Breyre, Bennett Lee, Nick R. Aldridge, Karl A. Sporer, Kristi L. Koenig, Marianne Gausche-Hill, Angelo A. Salvucci, Eric M. Rudnick, John F. Brown, Gregory H. Gilbert

**Affiliations:** * University of California, San Francisco, Department of Emergency Medicine, San Francisco, California; † University of Arizona, Department of Emergency Medicine, Tucson, Arizona; ‡ Alameda Health System, Highland Hospital, Department of Emergency Medicine, Oakland, California; § Hawaii Emergency Physicians Associated, Kailua, Hawaii; ¶ Kaiser Permanente San Diego, Department of Emergency Medicine, San Diego, California; || County of San Diego Health & Human Services Agency, EMS, University of California, Irvine, Department of Emergency Medicine, Orange, California; # Harbor-UCLA Medical Center, Department of Emergency Medicine, Los Angeles County EMS Agency, Santa Fe Springs, California; ** Santa Barbara EMS agency, Santa Barbara, California; †† Northern California EMS Agency, Redding, California; ‡‡ Stanford University, Department of Emergency Medicine, Palo Alto, California

## Abstract

**Introduction:**

We developed evidence-based recommendations for prehospital evaluation and treatment of adult patients with respiratory distress. These recommendations are compared with current protocols used by the 33 local emergency medical services agencies (LEMSA) in California.

**Methods:**

We performed a review of the evidence in the prehospital treatment of adult patients with respiratory distress. The quality of evidence was rated and used to form guidelines. We then compared the respiratory distress protocols of each of the 33 LEMSAs for consistency with these recommendations.

**Results:**

PICO (population/problem, intervention, control group, outcome) questions investigated were treatment with oxygen, albuterol, ipratropium, steroids, nitroglycerin, furosemide, and non-invasive ventilation. Literature review revealed that oxygen titration to no more than 94–96% for most acutely ill medical patients and to 88–92% in patients with acute chronic obstructive pulmonary disease (COPD) exacerbation is associated with decreased mortality. In patients with bronchospastic disease, the data shows improved symptoms and peak flow rates after the administration of albuterol. There is limited data regarding prehospital use of ipratropium, and the benefit is less clear. The literature supports the use of systemic steroids in those with asthma and COPD to improve symptoms and decrease hospital admissions. There is weak evidence to support the use of nitrates in critically ill, hypertensive patients with acute pulmonary edema (APE) and moderate evidence that furosemide may be harmful if administered prehospital to patients with suspected APE. Non-invasive positive pressure ventilation (NIPPV) is shown in the literature to be safe and effective in the treatment of respiratory distress due to acute pulmonary edema, bronchospasm, and other conditions. It decreases both mortality and the need for intubation. Albuterol, nitroglycerin, and NIPPV were found in the protocols of every LEMSA. Ipratropium, furosemide, and oxygen titration were found in a proportion of the protocols, and steroids were not prescribed in any LEMSA protocol.

**Conclusion:**

Prehospital treatment of adult patients with respiratory distress varies widely across California. We present evidence-based recommendations for the prehospital treatment of undifferentiated adult patients with respiratory distress that will assist with standardizing management and may be useful for EMS medical directors when creating and revising protocols.

## INTRODUCTION

Adults with respiratory distress make up 6–12% of all patients transported by emergency medical services (EMS).^1^–^3^ This subgroup of EMS patients is older and sicker than other transported patients and patients who arrive to the ED by other transport methods. A study of adults with dyspnea in Australia and New Zealand showed the average age is 74 years, 76% are admitted, 6% are intubated, and 6% of admitted patients do not survive to hospital discharge.^4^–^6^ Three diagnoses (pneumonia, heart failure, and chronic obstructive pulmonary disease [COPD] exacerbation) account for 60% of cases.^4^

EMS personnel play a prominent role in triage, transport, and initial management of adult patients with respiratory distress. For these patients, Stiell et al demonstrated that, compared with Basic Life Support, Advanced Life Support-level prehospital care results in a decrease of mortality to 12.4% from 14.3% and a substantial improvement in symptom relief due to early therapeutic interventions.^6^ The delivery of early, targeted therapy by paramedics is often hindered by the diagnostic challenge of respiratory distress. Diagnostic accuracy of paramedics in patients with acute dyspnea has been shown to vary between 53% and 77%.^4^,^5^,^7^–^9^ They perform better in patients with asthma or COPD and worse in patients with acute pulmonary edema (APE).^4^,^5^,^7^,^10^

Without widely accepted guidelines, EMS care continues to vary greatly across the United States. In 2007 the Institute of Medicine report, “Emergency Medical Services at the Crossroads,” advocated for the development of evidence-based model prehospital protocols so that all patients would receive the current standard of care. Therefore, we aim to provide a summary of the evidence for prehospital treatment of adult patients with respiratory distress, and to assess the consistency of California protocols with respect to our recommendations.

## METHODS

The state of California divides EMS care into 33 local EMS agencies (LEMSA). Each of these geographically divided governmental regulatory bodies has a set of medical control protocols in accordance with California EMS Authority scope of practice. Medical directors of those agencies, along with other EMS medical directors, make up the EMS Medical Directors Association of California (EMDAC). EMDAC supports the various agencies and makes recommendations to the California EMS Authority about policy, legislation, and scope of practice issues. In an effort to improve quality and decrease variability in EMS practice in California, EMDAC has endeavored to create evidence-based recommendations for EMS protocols.^11^–^14^

A subcommittee of EMDAC, the Medical Advisory Committee, chose the elements that should be included in any protocol for an adult patient with respiratory distress. Searches of MEDLINE, MEDLINE Scopus, Web of Science, and the Cochrane Database were performed. All searches were limited to English-language sources, adults, and human studies. In addition, relevant articles from the bibliographies of included studies and more recent emergency department (ED) and prehospital articles identified by committee members and reviewers were included. When there was minimal prehospital research, the most pertinent ED data was reviewed.

**Population Health Research Capsule**

What do we already know about this issue?

*Adults with respiratory distress make up 6–12% of EMS transports and are older, sicker, and have a high mortality. Prehospital care has demonstrated a decrease in mortality*.

What was the research question?

*An evidence-based review will highlight treatments that benefit these patients and demonstrate areas that need more research*.

What was the major finding of the study?

*Reducing the variability and optimizing the prehospital care of the adult respiratory patient will decrease medical costs and improve survival*.

How does this improve population health?

*Nitrates in patients with acute pulmonary edema are likely helpful but have poor quality research to support them*.

Additionally, the references of included papers were examined for additional studies. The interventions that were found in published prehospital and ED studies were then used to create clinical questions using the population, intervention, control group and outcome (PICO) format. Recommendations, based on the studies found, were created for each PICO question.

The Medical Advisory Committee assigned levels of evidence (LOE) and graded their recommendations based on a tailored modification of the American College of Emergency Physicians clinical policymaking process.^15^ LOE (Table 1) were assigned based on the study design, including features such as data collection methods, randomization, blinding, outcome measures and generalizability. (A brief summary of the reviewed studies is available in an electronic appendix.) After assigning LOE to the studies, these were translated to clinical grades of our recommendations using the standards described in Table 2.

In January 2019, we reviewed the protocols of all 33 LEMSAs for comparison with our recommendations. We deemed institutional review board approval not necessary for this review of publicly available research and clinical protocols.

## RESULTS

**PICO Question:** Does the titration of oxygen in patients with respiratory distress improve outcomes?

### Summary of Current Evidence

In both hospital and prehospital care, oxygen is among the most common therapies administered to patients. Excess oxygen, however, has been linked to central nervous system toxicity, coronary vasoconstriction, and acute lung injury.^16^ A number of publications and recommendations have addressed oxygen use and titration in medically and surgically ill adults.^16^–^19^

Chu et al published one of the largest systematic reviews in *Lancet* using 25 randomized control trials that enrolled 16,037 patients with sepsis, critical illness, stroke, trauma, myocardial infarction, cardiac arrest, and patients who required emergency surgery.^16^ They compared patients receiving a liberal oxygen strategy (median fraction of inspired oxygen [FiO_2_] of 0.52) with a conservative oxygen strategy (median FiO_2_ of 0.21). The study showed that patients treated with a liberal oxygen strategy had increased in-hospital mortality (relative risk [RR] 1.21; confidence interval [CI] 1.03–1.43) and 30-day mortality (RR 1.14; CI 1.01–1.29) but showed similar morbidity. The authors concluded that supplemental oxygen may be harmful above peripheral capillary oxygen saturation (SpO_2_) 94–96%.^16^

Following the results published by Chu et al, the *BMJ* published clinical practice guidelines on oxygen management.^17^ For patients receiving supplemental oxygen, it was recommended to aim for SpO_2_ no more than 96%. For patients with acute myocardial infarction and stroke, it was recommended to not start supplemental oxygen for SpO_2_ greater than or equal to 93% (strong recommendation, or greater than or equal to 90–92%, weak recommendation). The authors also recommend that a target SpO_2_ range of 90–94% seems reasonable for most patients and 88–92% for patients at risk of hypercapnic respiratory failure. Excluded are patients who require a higher oxygen target closer to 100% to treat an underlying medical condition such as pneumothorax, carbon monoxide poisoning, cluster headache, and sickle cell crisis.

### Recommendation

#### Level B Recommendation

For patients who are receiving oxygen for respiratory distress, oxygen should be titrated to target SpO_2_ no more than 94–96%. This does not apply to those patients for whom 100% oxygen is the treatment of the underlying disorder or for those who are being preoxygenated prior to advanced airway placement.

**PICO Question:** Does the prehospital titration of oxygen to patients with suspected COPD improve outcomes?

### Summary of Current Evidence

A number of retrospective studies have demonstrated worse outcomes in patients with acute exacerbations of COPD treated with excessive oxygen such as higher rates of death, respiratory failure,^20^ or increased rates of respiratory acidosis.^21^ A prehospital, cluster-randomized, controlled, parallel group trial of oxygen therapy in patients aged 35 years or older with suspected bronchospasm was performed.^22^ It compared titrated oxygen (SpO_2_ of 88–92%) to high flow oxygen regardless of SpO_2_. Titrated oxygen treatment significantly reduced mortality, hypercapnia, and respiratory acidosis compared with high flow oxygen in acute exacerbations of COPD.

### Recommendation

#### Level A Recommendation

In prehospital patients with COPD exacerbations, oxygen should be titrated to a target of 88–92%.

**PICO Question:** In patients with suspected bronchospasm (asthma or COPD) in the prehospital environment, does prehospital administration of steroids have a benefit?

### Summary of Current Evidence

Characterized by respiratory distress and wheezing, asthma and COPD are both diseases of pulmonary obstruction. They often are both treated in EMS using protocols for bronchospasm. In examining the literature supporting steroid use, however, the disease entities are usually studied separately.

### Asthma

A meta-analysis by Rowe et al examined studies on the administration of steroids during an asthma exacerbation and its effect on pulmonary function, admission rates, and relapse rates.^23^ While having an equivalent effect on pulmonary function, steroids were effective at preventing relapse (odds ratio [OR] 0.15; Cl 0.05–0.44) and admissions in adults (OR 0.47; 95% CI 0.27–0.79) and children. The authors concluded that steroids were an important part in the emergency treatment of asthma exacerbations.

In 1999, Lin et al published a randomized, double blind, controlled trial exploring the effect of 125 milligrams (mg) of intravenous (IV) methylprednisolone vs placebo in 60 patients who failed to completely respond after one nebulized albuterol treatment.^24^ They found that patients who received methylprednisolone showed statically greater improvement in pulmonary function, and an improvement that occurred faster than the control group. They concluded that steroids should be given early in the course of treatment of patients with asthma exacerbations.

A subsequent Cochrane review by Rowe et al in 2001 examined studies looking at steroids in asthma treatment on the primary outcome of admission rates.^25^ They included 12 studies in their analysis and found that when steroids were received within one hour of arrival to the ED, there was decrease in admission rates. This effect was first present two hours after steroid administration and most pronounced between 4–6 hours after administration.

In an attempt to explore whether the effect of systemic steroids extended to the prehospital arena, Knapp et al published a retrospective case review comparing admission rates in patients with moderate to severe asthma exacerbations who received 125 mg IV methylprednisolone via EMS compared to in the ED.^26^ They found that patients who received steroids via EMS had a lower admission rate (13% compared to 33%) and had a quicker resolution of symptoms (15 +/− 7 minutes compared to 40 +/− 22 minutes).

### Chronic Obstructive Pulmonary Disease

In 2014, a Cochrane review by Walters et al examined the effect of systemic steroids on acute exacerbations of COPD.^27^ They identified 16 studies comparing orally or parenterally administered steroids with placebo in COPD treatment. While there was no mortality difference, they found high quality evidence that systemic steroids reduced the likelihood of treatment failure by over half (OR 0.48; CI 0.35–0.67). There was also moderate quality data that systemic steroids reduced the rate of relapse by one month and reduced total hospital length of stay in admitted patients. It also found that route of administration (parenteral vs oral) did not lead to any difference in primary outcomes of treatment failure, relapse, mortality, or any secondary outcome.^27^ This has been demonstrated by other studies as well. Lindenauer et al demonstrated oral low-dose steroids did not result in worse outcomes compared to high-dose IV steroids among hospitalized patients with COPD exacerbations.^28^

We identified no prehospital studies that explored the use of steroids in patients with COPD exacerbations. The Cochrane review noted that about 1 in 6 patients experience an adverse effect from corticosteroid administration: the most common of these was hyperglycemia. This was higher in those doses given parenterally. There was a non-significant increase in psychiatric disturbance. Intensive care unit studies did not show significant increase in gastrointestinal bleeding, or hypertension.^28^

### Recommendation

#### Level B Recommendation

In patients with suspected bronchospasm (asthma or COPD), systemic steroids (by mouth or IV) should be administered in the prehospital environment.

**PICO Question**: Does the prehospital administration of albuterol to patients with suspected bronchospasm improve outcomes?

### Summary of Current Evidence

The studies looking at the use of albuterol in patients with respiratory distress and suspected bronchospasm are limited.^29^–^34^ Many prehospital observational studies demonstrate the safety of prehospital use of nebulized albuterol and improvements in subjective symptoms and peak expiratory flow rates. The available literature becomes slightly more expansive when including other beta-2 agonists such as levalbuterol,^31^,^35^ salbutamol,^30^ and terbutaline.^30^,^36^

In one large observational cohort study of 3351 prehospital patients, patients demonstrated significant improvement in reported dyspnea and peak flow rates.^37^ In a different retrospective study, prehospital administration of nebulized albuterol did not affect travel interval, length of stay in the ED, or medication use after ED presentation. 33

One prehospital randomized double-blind trial studied asthma patients receiving either subcutaneous terbutaline or nebulized albuterol.^36^ This small study of 83 patients demonstrated a greater improvement in respiratory distress visual analog scale scores than did the terbutaline group. Hospital admission rates, vital signs, and peak expiratory flow rates were not significantly different.

### Recommendation

#### Level B Recommendation

In patients with suspected bronchospasm (asthma or COPD), albuterol should be administered in the prehospital environment.

**PICO Question:** Does the prehospital administration of ipratropium to patients with suspected bronchospasm improve outcomes?

### Summary of Current Evidence

There is weak evidence from the ED management of acute asthma that ipratropium improves airflow obstruction and possibly reduces hospital admissions when used as an adjunct to beta 2 agonists.^38^–^40^ A single before-and-after analysis of the addition of ipratropium to albuterol was the only identified prehospital study.^41^ It found no differences in outcomes as compared to albuterol alone.

### Recommendation

#### Level C Recommendation

In patients with suspected bronchospasm, ipratropium can be administered; however, there is limited data from the prehospital setting. The benefits are greatest in confirmed asthmatics and in those having a severe exacerbation.

**PICO Question:** In patients with suspected acute pulmonary edema, does prehospital use of nitroglycerin have a benefit?

### Summary of Current Evidence

A number of case series and retrospective studies have demonstrated the clinical effects of nitroglycerin in patients with suspected APE. Nitroglycerin is a potent vasodilator that improves hemodynamics by decreasing pulmonary arterial pressure and reducing left ventricular preload and afterload.^42^,^43^

A randomized trial by Cotter et al examined patients with severe pulmonary edema who received either a high dose of isosorbide dinitrate and a low dose of furosemide vs a low dose of isosorbide dinitrate and a high dose of furosemide. With 52 patients in each arm they found that patients who were randomized to receive a higher dose of nitrates had a lower rate of mechanical ventilation (13% vs 40%), myocardial infarction (17% vs 37%) and death (1.9% vs 5.8%).^44^

In a secondary analysis of a multicenter randomized controlled trial (RCT) of ED non-invasive ventilation vs oxygen, Gray et al aimed to examine the effect of diuretics, nitrates, and opiates on patients with severe pulmonary edema.^45^ The study concluded that there was no evidence that nitrates were associated with any difference in mortality, improvement in acidosis or respiratory distress. The authors suggest these findings may reflect that nitrates are most effective when given to patients with pulmonary edema and hypertension.^45^

There are several thoughtful reviews regarding prehospital care nitrates. In a 2003 review, the authors conclude by consensus that high-dose nitrates represent the out-of-hospital treatment of choice for APE.^42^ They outline prehospital treatment that uses parameters such as systolic blood pressure and severity of symptoms to guide nitrate treatment.

Overall the evidence on prehospital use of nitrates is limited and at times conflicting. An important theme in the literature is the high rate of misdiagnosis of APE and the implications of incorrect administration of nitrates. If nitrates are to be used prehospital, there should be clearly defined parameters, for example systolic blood pressure minimums (90 millimeters of mercury), that might help target APE patients who would benefit most from the effects of nitrates.^11^

### Recommendation

#### Level C Recommendation

In patients with APE in the prehospital environment, administration of nitrates may be beneficial in critical, hypertensive patients. The ability to correctly diagnose prehospital APE may limit potential benefits of nitrates.

**PICO Question:** In patients with suspected APE, does prehospital use of furosemide have a benefit?

### Summary of Current Evidence

Furosemide is frequently used in the treatment of congestive heart failure. The diuretic effect helps decrease total body fluid volume, which can decreasing left ventricular filling pressure.^46^ A 1987 prospective study examined medication treatment of 57 prehospital patients with presumed APE.^47^ Outcomes included subjective patient responses, vital sign improvement, scaled respiratory distress evaluation, and adverse effects. Investigators concluded that furosemide does not add to the efficacy of treatment for presumed prehospital APE and may be in fact deleterious; cases of hypotension and hypokalemia were noted, and 25% of patients later required fluid resuscitation. Despite its small sample size this is the only prospective study identified in the review of current literature.

A retrospective chart review in 2006 identified 144 patients who received prehospital furosemide for presumed APE.^48^ Investigators found the rate of misdiagnosis high at 41%. Furosemide was administered when it was not indicated in 42% of patients and potentially harmful in 17% of patients, such as those with sepsis due to pneumonia. Given the high prevalence of inappropriate and harmful administration of furosemide, the investigators advised against prehospital diuretic use.

Overall the evidence on prehospital furosemide for APE is limited. An important finding in the literature is the rate of misdiagnosis of APE and the implications that can have for incorrect administration of furosemide.

### Recommendation

#### Level C Recommendation

In patients with APE in the prehospital environment, there is insufficient evidence to demonstrate that furosemide may be beneficial.

#### Level B Recommendation

There is moderate evidence to support that prehospital furosemide administration may be harmful, particularly when patients are incorrectly diagnosed with APE.

**PICO Question**: In patients with respiratory failure, does prehospital use of non-invasive positive pressure ventilation (NPPV) have a benefit? Is there benefit in those with APE? Is there benefit in those with bronchospasm?

### Summary of Current Evidence

NPPV provides ventilatory assistance to those in respiratory distress by supporting both oxygenation and ventilation.^49^,^50^ Use of NPPV has steadily increased in the ED, and a number of randomized trials and meta-analyses have evaluated its safety and effectiveness to assist those patients with severe respiratory distress and hypoxia from an acute asthma exacerbation,^50^,^51^ APE,^49^,^52^–^54^ or undifferentiated respiratory distress.^55^ These studies have generally found earlier improvement of respiratory distress, vital signs, and metabolic abnormalities.^55^ There is moderate evidence that NPPV lowers the rate of intubation. A number of these studies have also demonstrated a mortality benefit.

NPPV, primarily continuous positive airway pressure (CPAP) due to equipment limitations, gained traction in EMS in the late 1990s. Current models create pressure from a positive end-expiratory pressure valve or adjusting the amount of oxygen going to the device. Early prehospital retrospective studies demonstrated safety and likely clinical improvements.^56^–^59^ Studies have also examined the effectiveness of NPPV on the treatment of an acute COPD exacerbation,^57^ APE,^56^,^58^,^60^,^61^ and undifferentiated significant respiratory distress.^59^,^62^–^66^

A prospective, non-blinded RCT looking at the use of prehospital CPAP for patients with acute respiratory failure compared with standard care found that intubations decreased by 30% and mortality decreased by 21%.^62^ Although the study included a relatively small number of patients, the clinical outcome was significant.

A subsequent systematic review and meta-analysis focused on studies examining prehospital CPAP and its effect on intubations and mortality in patients with acute respiratory failure.^64^ Three RCTs, one non-randomized comparative study, and one retrospective chart review included 1002 patients and found significantly fewer intubations (OR 0.31; 95% CI 0.19–0.51) and lower mortality (OR 0.41; 95% CI 0.19–0.87) with CPAP use.^60^–^66^

### Recommendation

#### Level A Recommendation

There is sufficient evidence that demonstrates the safety and benefit of non-invasive ventilation (primarily CPAP) in those patients with undifferentiated respiratory distress.

#### Level A Recommendation

There is sufficient evidence that demonstrates the safety and benefit of non-invasive ventilation (primarily CPAP) in those patients with suspected APE.

#### Level A Recommendation

There is sufficient evidence that demonstrates the safety and benefit of non-invasive ventilation (primarily CPAP) in those patients with suspected respiratory distress due to bronchospasm.

### Comparison with 33 Local EMS Agency Protocols

All 33 LEMSAs had at least one protocol for the prehospital management of respiratory distress as shown in Table 3.

## DISCUSSION

There is a paucity of research on specific prehospital practices used in managing respiratory distress. Hospital-based studies can inform the development of EMS protocols, but limitations such as provider skills, diagnostic ability, time, and scene dynamics make direct correlation impractical. Whenever possible, prehospital studies are preferred. A major theme of the prehospital literature is the diagnostic challenge undifferentiated respiratory distress presents. Inappropriate use of nitroglycerin or furosemide has the potential to be harmful. However, the benefit of NPPV for several etiologies of respiratory distress is well supported.

The respiratory distress protocols reviewed varied greatly in content and structure between LEMSAs in California, reflecting the variation between states.^11^–^14^ Goal SpO_2_ and O_2_ titration varied widely. Seventeen agency protocols include a lower limit of acceptable SpO_2_ before oxygen is to be administered, and three protocols recommended further titration after oxygen is applied. This is reasonable given that supplemental oxygen is intended to treat hypoxemia and has not been shown to consistently relieve breathlessness in the absence of hypoxemia.^18^ For those patients with COPD, only four protocols called for lower SpO_2_ goals. Current literature and guidelines reinforce that liberal oxygen administration is not benign and should be dosed appropriately. Adjusting current SpO_2_ targets for patients in respiratory distress should be relatively easy to implement. While this adjustment would likely increase the attention needed to avoid overand under-oxygenation, titration would need no new equipment, use less overall oxygen, and likely be more comfortable for the patient. As stated above, this recommendation for titration does not apply to those patients for whom oxygen is the treatment for the underlying condition such as pneumothorax and carbon monoxide poisoning.

Albuterol is recommended by all LEMSAs while ipratropium is only prescribed by 15. The evidence supporting prehospital ipratropium is weaker than for albuterol in patients with exacerbations of COPD and asthma. These conditions are relatively easier to diagnose in the prehospital environment since both chronic conditions are prevalent and patients tend to be familiar with their own symptoms.

Currently, steroids are not administered by EMS in California for bronchospasm. The literature reviewed supports its introduction for the treatment of asthma and COPD as it helps in symptom resolution and reducing both relapse and hospital admissions. The most common side effect described was hyperglycemia in those patients with COPD, which is reduced by using oral steroids. Oral administration (most commonly 60 mg prednisone) was found to be as effective as parenteral steroid administration (most commonly 135 mg IV methylprednisolone).

Nitroglycerin is prescribed by every LEMSA but there are significant variations in dosages, treatment intervals, and blood pressure parameters. The variation in dosing mimics the variation often found in EDs, with recent data demonstrating the use and safety of higher loading doses of nitroglycerin.^67^

Only one LEMSA included furosemide in the treatment of APE. The research found did not support widespread use of furosemide outside of the hospital. The protocol appears to have been written for a rural environment and requires base hospital contact prior to medication administration as well as a transport time exceeding 45 minutes.

Non-invasive ventilation, CPAP, is present in the protocols of every LEMSA for the treatment of APE and bronchospasm. CPAP is also indicated for undifferentiated respiratory distress in most protocols.

## LIMITATIONS

We analyzed the protocols of only one state; therefore, the protocol conclusions cannot be generalizable to other states. We did not contact the individual LEMSAs to learn about motivation for differences between protocols. There are always inherent biases when synthesizing available data into recommendations. Finally, many recommendations are at least partly derived from hospital-based studies because of a lack of adequate prehospital studies.

## CONCLUSION

Protocols for respiratory distress vary widely across the state of California. The evidence-based recommendations created via GRADE methodology (Grading of Recommendations Assessment, Development and Evaluation) for the prehospital management of this condition may be useful for EMS medical directors tasked with creating and revising these protocols.

## Supplementary Information



## Figures and Tables

**Table 1:** Level of evidence definitions.

LOE Level	Definition
I	Randomized, controlled trials, prospective cohort studies, meta-analysis of randomized trials or prospective studies, or clinical guidelines/comprehensive review.
II	Nonrandomized trials and retrospective studies.
III	Case series, case reports, and expert consensus.

*LOE*, levels of evidence.

**Table 2:** Recommendation definitions.

Level Recommendation	Definition
A	Prehospital recommendations with a strong degree of certainty based on one or more LOE I studies or multiple LOE II studies.
B	Prehospital recommendations with a moderate degree of certainty based on one or more LOE II studies or multiple LOE III studies.
C	Prehospital recommendations based on only poor quality or minimal LOE III studies or based on consensus.

*LOE*, levels of evidence.

**Table 3:** The protocols of the 33 Local EMS Agencies (LEMSAs) in California were examined regarding specific treatments in the care of patients with respiratory distress. There is variability among the different agency protocols. This is most pronounced in the titration of oxygen for patients with and without COPD.

**Titration of oxygen in patients with respiratory distress (to no more than 96%)** varied significantly among protocols: 21 LEMSAs included either oxygen titration or an acceptable lower limit of normal prior to oxygen administration, most commonly 94%.
**Titration of SpO_2_ in COPD was recommended in** three LEMSAs ranging from 88–92% to 92–94%. One LEMSA recommended reduced oxygen but did not provide a goal SpO_2_.
**Administration of albuterol in suspected bronchospasm** was included in all LEMSAs.
**Administration of ipratropium in suspected bronchospasm** was included in 15 LEMSAs.
**Administration of nitroglycerin in suspected APE** was included in all LEMSAs but varied in the dosing, titration parameters, and contraindications. A single 0.4 mg sublingual tablet was the most common initial dose and form of the medication. Eight protocols included instructions for nitroglycerin paste and one included nitroglycerin spray in addition to the tablets. The minimum systolic blood pressure varied between 90 and 100 mmHg. Eleven protocols noted that nitroglycerin administration is contraindicated if a patient is taking phosphodiesterase inhibitors.
**Administration of furosemide in suspected APE** was only included in one protocol.
**The use of NPPV for acute pulmonary edema** was included in all LEMSAs.
**The use of NPPV for bronchospasm** was included in all LEMSAs.
**The use of NPPV for undifferentiated respiratory distress** was included in 26 LEMSAs.

*COPD*, chronic obstructive pulmonary disease; *APA*, acute pulmonary edema; *NPPV*, non-invasive positive pressure ventilation.
