# Supplementary material for: Adult Patients with Respiratory Distress: Current Evidence-based Recommendations for Prehospital Care
Source: West J Emerg Med. 2020 Jun 25;21(4):849–57. doi: 10.5811/westjem.2020.2.43896 (PMC7390576; doi:10.5811/westjem.2020.2.43896)
Supplement: Supplementary file 1 [file wjem-21-849-s001.docx]

**Appendix**

Evidentiary Table: Bronchospasm treatment

| **Study** | **LOE** | **Study Design** | **Methods and Outcomes** | | **Results** | **Limitations** | **Overall** |
| --- | --- | --- | --- | --- | --- | --- | --- |
| **Nebulized Albuterol** | | | | | | | |
| Delbridge et al. 2003 |  | Review Article | |  |  |  |  |
| Campbell et al. 1995 | II | Prospective comparison | | Patient broken into 3 districts (A, B, C) and patient’s given treatments based on district. A – 5mg terbutaline nebuhaler, B – 5mg salbutamol nebulizer, C – 200 ug salbuterol MDI  Outcomes – RR, pulse rate, peak expiratory flow rate, and subjective assessment of severity of breathing. Repeat 30 minutes later or on arrival to hospital. | Nebulized albuterol improved respiratory rate and breathlessness score. | No spacer device used with inhaler  Patient not randomized, broken into districts  Salbutamol – more ptent than terbutaline | Nebulized albuterol is treatment of choice in prehospital compared to MDI/neb terbutaline |
| Rodenberg H et al. 2002 | IV | Open label medication administration | | Examined effect of levalbuterol on prehospital patent parameters. No comparison  Pulse, RR, Respiratory distress, Peek Expiratory Flow | Improvement in all parameters without significant tachycardia | Open label, no comparison | Prehospital Levalbuterol improves RR, Respiratory distress and PEF w/out significant effect on HR |
| Wathen CG et al. 1990 | IV | QI analysis | | Salbutamol added to prehospital service  Examined respiratory symptoms | Improvement in 80% of patients | QI not research | Salbutamol led to symptom improvement in asthmatics |
| Weiss SJ et al. 1994 | IV | QI analysis | | Pre/Post analysis of the addition of albuterol on transport time and ED morbidity | Prehospital albuterol increased scene time (9.7 vs 12.1 min) and first post treatment PEF rate. There was a decrease in initial patient severity.  There was no effect on travel interval, ED LOS, or medication in ED | Older study, 1994  Pre/Post analysis | Albuterol administration increases scene time but decreases severity by the time of ED arrival |
| Dickinson ET et al. 1992 |  | Prospective comparison | | Pre/Post comparison of 62 patients receiving nebulized albuterol for shortness of breath and wheezing on exam. Examined peak expiratory flow rates (PEFR) and subjective dyspnea. | Overall duration of treatment was 9.4 minutes, subjective improvement was reported by 95% of patients. Improvement in peak flow was noted to be statistically significant in patient with asthma and COPD. | Older study, 1992  Pre/Post analysis | Nebulized albuterol improves PEFR and subjective dyspnea. |
| Thompson M et al. 2004 |  | Prospective comparison | | Pre/Post comparison of 196 patients. Outcome variable as change in PEFR after single EMS treatment with levalbuterol or albuterol. | Both albuterol and levalbuterol produced an improvement from peak flow rates from baseline (p< 0.001) however there was no difference noted between groups. | Pre/Post analysis  Open label study | Both albuterol and levalbuterol improve peak flow rate. |
| Zehner WJ et al. 1995 | I | Double placebo, double blind, randomized trial | | Compared subq terbutaline with nebulized albuterol  Examined respiratory severity (score based on RR, wheezing, speech, PEF) and patient rating of their own resp distress | Respiratory severity (score based) was not significantly different between terbutaline and albuterol. Albuterol provided greater subjective improvement | Comparison is subcutaneous terbutaline, not currently used commonly in prehospital. | Albuterol and terbutaline improved respiratory distress. Albuterol performed better with subjective symptom resolution of the patient. |
| **Nebulized Ipratropium** | | | | | | | |
| Rodrigo G et al. 1990 | I | Systematic Review and Meta-analysis | | Selected only randomized, double-blind controlled trials of patients with acute asthma exacerbation evaluating for effect of ipratropium  Examined admission rates and pulmonary function | Modest improvement in pulmonary function and significant reduction in admission rates | heterogeneity | Improvement in pulmonary function and reduction of admission rates |
| Aaron SD et al. 2001 | I | Systematic Review and meta-analysis | | Only randomized, placebo-controlled studies.  Adults with acute asthma presenting to ED or acute care facility.  Examined FEV1 and PEF  Unable to examine ED LOS, admission rate | Improvement of FEV1 and PEF. Greater effect in more severe exacerbations | Unable to assess important patient centered outcomes like admission/ED LOS.  Heterogeneity | Ipratropium has a modest effect on improving FEV1 and PEF. The effect is greater on those with more severe exacerbations |
| Cydulka RK et al. 2010 | I | Prospective, double-blind, randomized controlled study | | Levalbuterol vs Levalbuterol + Ipratropium  Changes in FEV1 at 30 and 60 min.  Hospitalization rate, relapse rate | No significant difference in FEV 1 at 30 or 60min.  No difference in hospitalization rate.  Possible increase in side effect (palpitation) with ipratropium | Only 2 centers  High rate of smokers. | Ipratropium does not add superiority in improving FEV1 or decreasing hospitalization |
| Davis DP et al. 2009 | II | Before and after study (retrospective chart review) | | Prior to July 1, 2000, only getting albuterol neb (2.5mg), after getting albuterol/ipratropium (2.5mg/0.5mg)  Reviewed data of dyspnea patients 6 months prior to adding ipratropium to albuterol neb and 6 months after.  Main outcome: need for hospital admission.  Secondary outcomes: HR, BP, RR, SaO2, and Improvement of clinical course | No difference in admission rate  No difference in HR, BP, RR, SaO2, or clinical course | Retrospective chart review and patients not randomized  Heterogeneous patient group (all patients with acute exacerbation of reactive airway disease)  Did not examine treatments in ED, unable to get PFTs | No difference between albuterol alone vs albuterol/ ipratropium in prehospital setting |

**REFERENCES:**

1. Delbridge T, Domeier R, Key CB. Prehospital asthma management. *Prehosp Emerg Care.* 2003;7(1):42-47.

2. Campbell IA, Colman SB et al. *An open, prospective comparison of B2 agonists given via nebulizer, Nebuhaler, or pressurized inhaler by ambulance crews as emergency treatment.* Thorax. 1995. 50. 79-80.

3. Rodenberg H. Effect of levalbuterol on prehospital patient parameters. *Am J Emerg Med.* 2002;20(5):481-483.

4. Wathen CG, Crompton GK, Carrington D, Hollingworth J. Treatment for acute asthma in the ambulance. *Br J Gen Pract.* 1990;40(338):388.

5. Weiss SJ, Anand P, Ernst AA, Orgeron D, May WL. Effect of out-of-hospital albuterol inhalation treatments on patient comfort and morbidity. *Ann Emerg Med.* 1994;24(5):873-878.

6. Dickinson ET, O'Connor RE, Megargel R. The prehospital use of nebulized albuterol on patients with wheezing whose chief complaint is shortness of breath. *Del Med J.* 1992;64(11):679-683.

7. Thompson M, Wise S, Rodenberg H. A preliminary comparison of levalbuterol and albuterol in prehospital care. *The Journal of emergency medicine.* 2004;26(3):271-277.

8.. Zehner WJ, Jr., Scott JM, Iannolo PM, Ungaro A, Terndrup TE. Terbutaline vs albuterol for out-of-hospital respiratory distress: randomized, double-blind trial. *Acad Emerg Med.* 1995;2(8):686-691.

9. Rodrigo G, Rodrigo C, Burschtin O. A meta-analysis of the effects of ipratropium bromide in adults with acute asthma. *The American journal of medicine.* 1999;107(4):363-370.

10. Aaron SD. The use of ipratropium bromide for the management of acute asthma exacerbation in adults and children: a systematic review. *The Journal of asthma : official journal of the Association for the Care of Asthma.* 2001;38(7):521-530.

11. Cydulka RK, Emerman CL, Muni A. Levalbuterol versuss levalbuterol plus ipratropium in the treatment of severe acute asthma. *The Journal of asthma : official journal of the Association for the Care of Asthma.* 2010;47(10):1094-1100.

12. Davis DP, Wiesner C et al. *The Efficacy of Nebulized Albuterol/Ipatropium Bromide versus Albuterol alone in the Prehospital Treatment of Suspected Reactive Airway Disease.* Prehospital Emergency Care. 2009. 9(4) 386-390.

Evidentiary Table: Acute Pulmonary (APE) Edema Treatment

| **Study** | **LOE** | **Study Design** | **Methods and Outcomes** | | **Results** | **Limitations** | **Overall** |
| --- | --- | --- | --- | --- | --- | --- | --- |
| **Nitroglycerin** | | | | | | | |
| Bertini et al. 1997^1^ | I | Retropsective | | Evaluation of prehospital records of mobile critical care unit comparing O2, morphine, furosemide, digoxin, nitrates, aminophylline or dopamine for CAPE  Outcome measured: mortality | Mortality rate decreased from 13% to 5.3% with IV nitrate use. | Choice of medication was at the discretion of physician. | IV Nitrates were effective in reducing mortality |
| Cotter et al^2^ | II | Randomized | | Emergency mobile unit randomized presumed CAPE. All treated with O2, furosemide, morphine and divided into two treatment protocols– A- isosorbide dinitrate, B- furosemide + high dose  Outcomes: Mechanical ventilation, myocardial infarction, death | Group A – 13% required mechanical  Group B- 40% required mechanical ventilation.  No significant difference in mortality between groups | No control group.  Small sample size. | Isosorbide dinitrate is safe and effective. |
| Gray et al. 2010 ^3^ | II | Analysis of data from 3CPO trial | | Analysis of data from 3CPO trial to see if individual interventions of nitrates, opiates, diuretics have an effect on patient outcomes.  Outcome measured: 7 day mortality, improvement of acidosis, improvement of respiratory distress | Opiates had less improvement of acidosis.  No difference in mortality, no difference in improvement of respiratory distress | Pt population was sick and acidotic – may not be generalizable to population  Majority of patients prescribed nitrates and diuretics (90% of pts) may be underpowered to see if there is a difference | Opiates have less improvement of respiratory distress 2/2 CHF  Nitrates diuretics did not change mortality or respiratory distress |
| Hoffman, Reynolds 1987 ^4^ | I | Prospective sequential trial | | Four treatment protocols for prehospital patients with presumed pulmonary edema. A – Nitroglycerin and furosemide. B – morphine and furosemide. C – morphine, nitro, furosemide  D – nitroglycerin and morphine  N = 57. (60 but 3 excluded  Not randomized due to resistance from EMS agencies  Each group had 15 sequentially entered patients (Group A then B and so on)  Measured vital signs, ED potassium, ED intubation, Acute MI, died in hospital. Also measured subjective improvement | Group A improved more than other groups.  No evidence of synergistic effect of meds.    Furosemide may be harmful. Dehydration and laboratory abnormailities (some required IV fluid resuscitation) | 23% of pts found not to have pulmonary edema  Small study  Measures were based on vital signs, not invasive measurement  Did not attempt to validated ED physicians diagnosis | Nitroglycerin is beneficial for prehospital pulmonary edema  Furosemide potentially harmful |
| Mosesso et al. 2003. ^5^ | III | Review | | Review analyzing the diagnosis and treatment strategies for acute congestive heart failure in prehospital setting | Nitrates are treatment of choice.  Morphine and furosemide for select patients.  CPAP is effective.  ACEi needs more data | Systematic review | Nitrates useful in the prehospital setting. CPAP is useful.  Morphine and furosemide may be useful.  ACEi not useful. |
| Wakai et al. 2013 ^6^ | I | Cochrane Systematic Review of RCTs | | Cochrane review of randomized control trials to compare nitrates (nitroglycerin, isosorbide dinitrate) to alternative interventions (furosemide + morphine, furosemide, hydralazine, prenalterol, IV nesiritide, and placebo) in treatment of acute heart failure syndrome  4 trials included  Outcomes: symptomatic relief, improvement of hemodynamics | No difference in time to symptomatic relief between nitroglycerin/NAC and morphine/furosemide  No evidence to support difference in requirement for mechanical ventilation, systolic BP, diastolic BP, pulmonary artery occlusion pressure, cardiac output, and progression to MI.  Higher adverse events with nitroglycerin compared to placebo at 3 hour | Limited number of studies (n=4)  Did not find consistent evidence to support a difference in acute heart failure patients treated with nitrates vs alternative interventions | Unclear if nitrates differ in effectiveness to alternative interventions in regards to symptomatic relief or hemodynamic variables |
| **Prehospital Furosemide** | | | | | | | |
| Hoffman et al ^4^ |  |  | | See nitrates section |  |  |  |
| Jaronik et al. 2006 ^7^ | II | Retrospective Chart Review | | Retrospective chart review to see if furosemide was given appropriately and if harm was done to the patients  Medication administration appropriate if diagnosis is CHF or pulmonary edema. Also if BNP>400.  Inappropriate if no diagnosis of CHF, BNP<400, or diagnosis of sepsis/dehydration/pneumonia | Furosemide appropriate in 58%.  Furosemide inappropriate in 42% of patients.  Furosemide potentially harmful in 17% of patients | Retrospective study. Based on prehospital documentation and may not be complete.  Did not account for bias due to pts well-known to EMS.  Did not account for EMS providers experience/ clinical abilities | Furosemide harmful when given in prehospital setting |
| Pan et al., 2015 ^8^ |  | Retrospective chart Review | | Multicenter review of EMS patients who received preshopsital furosemide for presumed CAPE  Outcomes: serious adverse events (acute renal failure, intubation, vasopressors, death) | Third of patients who received prehospital furosemide did not have CAPE.  No statistical significant association between furosemide and adverse events | Not randomized study. | Prehospital CAPE diagnosis is challenging |

Levels: I – strong evidence exists. II – fair evidence exists. III – weak evidence exists

**REFERENCES**

1. Bertini G, Giglioli C, Biggeri A, et al. Intravenous nitrates in the prehospital management of acute pulmonary edema. *Ann Emerg Med.* 1997;30(4):493-499.

2. Cotter G, Metzkor E, Kaluski E, et al. Randomised trial of high-dose isosorbide dinitrate plus low-dose furosemide versus high-dose furosemide plus low-dose isosorbide dinitrate in severe pulmonary oedema. *Lancet.* 1998;351(9100):389-393.

3. Gray A, Goodacre S, Seah M, Tilley S. Diuretic, opiate and nitrate use in severe acidotic acute cardiogenic pulmonary oedema: analysis from the 3CPO trial. *QJM.* 2010;103(8):573-581.

4. Hoffman JR, Reynolds S. Comparison of nitroglycerin, morphine and furosemide in treatment of presumed pre-hospital pulmonary edema. *Chest.* 1987;92(4):586-593.

5. Mosesso VN, Jr., Dunford J, Blackwell T, Griswell JK. Prehospital therapy for acute congestive heart failure: state of the art. *Prehosp Emerg Care.* 2003;7(1):13-23.

6. Wakai A, McCabe A, Kidney R, et al. Nitrates for acute heart failure syndromes. *Cochrane Database Syst Rev.* 2013(8):CD005151.

7. Jaronik J, Mikkelson P, Fales W, Overton DT. Evaluation of prehospital use of furosemide in patients with respiratory distress. *Prehosp Emerg Care.* 2006;10(2):194-197.

8. Pan A, Stiell IG, Dionne R, Maloney J. Prehospital use of furosemide for the treatment of heart failure. *Emerg Med J.* 2015;32(1):36-43.

Evidentiary Table: NIPPV treatment

| **Study** | **LOE** | |  | **Study Design** | **Methods and Outcomes** | **Results** | **Limitations** | **Overall** |
| --- | --- | --- | --- | --- | --- | --- | --- | --- |
|  | | **NIPPV** | | | | | | |

| Collins et al 2006 ^1^ | II | Meta-analysis | Meta-analysis identifying ED patients over 18 with APE, comparing standard  Endpoint mortality, intubation | Decrease in mortality and intubation rates | Metaanalysis  Heterogeneity (limited) | NPPV decreases mortality and intubation rates |
| --- | --- | --- | --- | --- | --- | --- |
| Dib et al 2012 ^2^ | III | Retrospective Chart Review | Retrospective chart review for pts w/ APE treated with CPAP in the field vs standard care  BP reduction, HR reduction, SpO2 improvement, Intubation reduction | Decrease in rates of intubation, blood pressure, HR and improvement of SpO2  CPAP is feasible in prehospital environment | Restrospective chart review | CPAP seems to be feasible and beneficial |
| Ducros et al 2011 ^3^ | I | Randomized Multicenter Trial | Prehospital CPAP vs standard care in mobile ICUs in patients with aucte CPE  Need for intubation and resolution of symptoms | Improved resolution and reduction of intubations at 2 hours | Non blinded | CPAP improves outcome more than usual care alone |
| Frontin et a 2011 ^4^ | I | Randomized trial | OOH pts w/ severe ACPE comparing optimal treatment (Lasix, oxygen, high-dose bolus of nitrate plus oxygen) vs optimal tx plus CPAP  Outcome treatment success (RR<25) O2 >90% | No difference in symptom resolution at 2 hours or death at 30 days | White European population. Non blinded | CPAP may not add benefit to optimal intravenous treatment |
| Gray et al 2008 ^5^ | I | Multicenter, open, prospective, randomized, controlled trial | Oxygen vs CPAP vs NPPV for patients with ACPE  Improvement at 1h, HR, Acidosis, Death/Intubation at 7 days | Improvement at 1 hour of dyspnea. No difference in combined death/intubation at 7 days | Question of whether intubation at 7 days is meaningful outcome | CPAP and NPPV leads to more rapid symptom improvement. No effect on short term mortality |
| Goodacre et al. 2014 ^6^ | I | Systematic review | Effectiveness of Prehospital CPAP or BiPAP in acute respiratory failure  Death, Intubation | CPAP reduces intubation and mortality. The effectiveness of prehospital BiPAP is less clear | Study heterogeneity | Prehospital CPAP is linked with reduced intubation and death.  There was no difference demonstrated with BiPAP. |
| Green et al 2017 ^7^ | III | Systematic Review | Systematic review of studies of adults with asthma treated with NPPV  “Outcomes” | Trend to better outcomes but too much variability | Excessive heterogeneity | No conclusive recommendaitons |
| Hubble et al. 2006 ^8^ | II | Nonrandomized group controlled trial | Non-randomized controlled group trial from 7/1/04 to 6/30/05 comparing CPAP + standard treatment (oxygen, nitrates, furosemide, morphine, and if needed ET tube) with standard treatment  Endpoint – intubation  Secondary outcome – mortality, hospital length of stay, and changes in physiologic variables ( | Standard treatment alone more likely to die.  CPAP had improvement of respiratory rate and dyspnea score compared to standard treatment | Non randomized.  Difficulty accurately diagnosing acute pulmonary edema in prehospital setting (paramedic accuracy is 76% - false positives often pneumonia or COPD)  Did not look at patients not diagnosed with acute pulmonary edema by paramedics (did not look at false negatives)  Control group got more nitrates and furosemide  Not controlled for hospital/ED interventions (4 different hospitals) | CPAP reduces intubation and decreases short term mortality |
| Keenan et al. 2004 ^9^ | I | Systematic Review of RCTs | Systematic reviews of RCTs (8 RCTs) to see the effect of CPAP on mortality and rate of intubation.  Outcome: mortality, rate of intubation | CPAP decreases rate of intubation  Unclear if it decreases mortality | Heterogeneity of patient populations | CPAP decreases rate of intubation |
| Knox et al. 2015 ^10^ | III | Retrospective Cohort Study | Retrospective chart review of patients in the mobile intensive care unit treated with CPAP vs pts not treated with CPAP  Outcomes: relationship between CPAP and intubations | Decreased rate of intubations in patients treated with CPAP | Data sets were 6 years apart (but no changes in training/protocol/equipment)  Ambiguity in paramedic reports  Data collection between two different sets was difficult | CPAP decreases rate of intubation for patients with acute respiratory distress in the prehospital setting |
| Mal et al. 2014 ^11^ | I | Systematic Review and Meta | Search for randomized controlled trials comparing OOH NPPV w/ standard therapy in adults with severe respiratory distress  Need for intubation, In-hospital mortality | Reduction in inhospital mortality and need for intubation | Clinical heterogeneity, no standard definition for conditions and standard treatment | Decrease in in-hospital mortality and need for intubation |
| Masip et al. 2005 ^12^ | I | Systematic review and meta-analysis | Search for parallel studies comparing NPPV to conventional therapy in pts with APE  Need for intubation, Mortality | NPPV decreases the need for intubation and mortality. Evidence better for CPAP than BiPAP | Lack of standard definition for condition. | Reduction in need for intubation and mortality |
| Schmidbauer et al. 2011 ^13^ | II | Prospective Randomized trial | Prehospital NIV in patients with COPD vs usual care  RR, Dyspnea, ICU length of stay | Improved dyspnea and length of stay | Non blinded, small sample size | Improves dyspnea and ICU LOS |
| Soroksky et al 2003 ^14^ | I | Prospective Randomized Placebo-controlled trial | Acute asthmatics, BiPAP vs usual care  Increase of FEV1, hospitalization | Improvement of FEV1 and hospitalization | Small size | BiPAP can alleviate asthma attacks faster, improve lung function and decrease hospitalization |
| Thompson et al. 2008 ^15^ | I | Prospective, Randomized, Nonblinded Control trial | RCT to compare usual care vs usual care with CPAP in the prehospital setting for **acute respiratory failure**  Inclusion:  Severe respiratory distress with failing respiratory effort (paramedic judgement), retractions, hypoxia, respiratory rate >25  Exclusions: respiratory arrest, apnea, RR<8, altered mental status, hypotension, cardiac ischemia, Age<16  Outcome: need for intubation  Secondary outcome: mortality, length of stay | Decreased percentage of patients who needed intubations. Decreased mortality | Small study, may be underpowered (n= 71, usual care = 35 (1 lost to follow up), CPAP = 36, (1 lost to follow up))  Unable to blind | CPAP decreases intubations, decreases mortality in acute respiratory failure |
| Williams et al. 2013 ^16^ | I | Systematic review and meta-analysis | Systematic review of 5 studies (3 RCT, 1 nonrandomized comparative study, and one retrospective chart review) to elicit the effect of CPAP in prehospital setting for patients with acute respiratory failure  Outcome: intubation rate, mortality rate | Reduction in intubations and mortality in patients who receive CPAP in prehospital setting | Had non randomized trials in data set  Data collection has risk of bias  All data came from developed countries and unclear if findings translate to developing world | Prehospital CPAP decreases intubations and mortality when used on patients with acute respiratory failure |

Levels: I – strong evidence exists. II – fair evidence exists. III – weak evidence exists

**REFERENCES**

1. Collins SP, Mielniczuk LM, Whittingham HA, Boseley ME, Schramm DR, Storrow AB. The use of noninvasive ventilation in emergency department patients with acute cardiogenic pulmonary edema: a systematic review. *Ann Emerg Med.* 2006;48(3):260-269, 269.e261-264.

2. Dib JE, Matin SA, Luckert A. Prehospital use of continuous positive airway pressure for acute severe congestive heart failure. *J Emerg Med.* 2012;42(5):553-558.

3. Ducros L, Logeart D, Vicaut E, et al. CPAP for acute cardiogenic pulmonary oedema from out-of-hospital to cardiac intensive care unit: a randomised multicentre study. *Intensive care medicine.* 2011;37(9):1501-1509.

4. Frontin P, Bounes V, Houze-Cerfon CH, Charpentier S, Houze-Cerfon V, Ducasse JL. Continuous positive airway pressure for cardiogenic pulmonary edema: a randomized study. *Am J Emerg Med.* 2011;29(7):775-781.

5. Gray A, Goodacre S, Newby DE, et al. Noninvasive ventilation in acute cardiogenic pulmonary edema. *N Engl J Med.* 2008;359(2):142-151.

6. Goodacre S, Stevens JW, Pandor A, et al. Prehospital noninvasive ventilation for acute respiratory failure: systematic review, network meta-analysis, and individual patient data meta-analysis. *Acad Emerg Med.* 2014;21(9):960-970.

7. Green E, Jain P, Bernoth M. Noninvasive ventilation for acute exacerbations of asthma: A systematic review of the literature. *Australian critical care : official journal of the Confederation of Australian Critical Care Nurses.* 2017;30(6):289-297.

8. Hubble MW, Richards ME, Jarvis R, Millikan T, Young D. Effectiveness of prehospital continuous positive airway pressure in the management of acute pulmonary edema. *Prehosp Emerg Care.* 2006;10(4):430-439.

9. Keenan SP, Sinuff T, Cook DJ, Hill NS. Does noninvasive positive pressure ventilation improve outcome in acute hypoxemic respiratory failure? A systematic review. *Crit Care Med.* 2004;32(12):2516-2523.

10. Knox N, Chinwe O, Themba N, Joseph F, Hormoz A. Relationship between intubation rate and continuous positive airway pressure therapy in the prehospital setting. *World J Emerg Med.* 2015;6(1):60-66.

11. Mal S, McLeod S, Iansavichene A, Dukelow A, Lewell M. Effect of out-of-hospital noninvasive positive-pressure support ventilation in adult patients with severe respiratory distress: a systematic review and meta-analysis. *Ann Emerg Med.* 2014;63(5):600-607 e601.

12. Masip J, Roque M, Sanchez B, Fernandez R, Subirana M, Exposito JA. Noninvasive ventilation in acute cardiogenic pulmonary edema: systematic review and meta-analysis. *Jama.* 2005;294(24):3124-3130.

13. Schmidbauer W, Ahlers O, Spies C, Dreyer A, Mager G, Kerner T. Early prehospital use of non-invasive ventilation improves acute respiratory failure in acute exacerbation of chronic obstructive pulmonary disease. *Emerg Med J.* 2011;28(7):626-627.

14. Soroksky A, Stav D, Shpirer I. A pilot prospective, randomized, placebo-controlled trial of bilevel positive airway pressure in acute asthmatic attack. *Chest.* 2003;123(4):1018-1025.

15. Thompson J, Petrie DA, Ackroyd-Stolarz S, Bardua DJ. Out-of-hospital continuous positive airway pressure ventilation versus usual care in acute respiratory failure: a randomized controlled trial. *Ann Emerg Med.* 2008;52(3):232-241, 241 e231.

16. Williams TA, Finn J, Perkins GD, Jacobs IG. Prehospital continuous positive airway pressure for acute respiratory failure: a systematic review and meta-analysis. *Prehosp Emerg Care.* 2013;17(2):261-273.

Evidentiary Table: Steroids for Asthma and COPD

| **Study** | **LOE** | **Study Design** | **Methods and Outcomes** | | **Results** | **Limitations** | **Overall** |
| --- | --- | --- | --- | --- | --- | --- | --- |
| **Steroids for bronchospasm** | | | | | | | |
| Chu DK et al. 2018^1^ | I | Systematic Review and Meta-analysis | | RCTs of patients with sepsis, critical illness, stroke, trauma, MI, cardiac arrest or required surgery. Liberal vs conservative oxygen  Examined mortality, morbidity | Liberal o2 associated with increased in-hospital mortality, mortality at 30 days and mortality at longest follow-up | Heterogentiy of data  Majority of patients cardiac/STEMI. ? relevance to general population | Liberal oxygen strategy associated with increased mortality |
| Siemieniuk RAC et al. 2018^2^ | III | Practice Guidelines | | Based on RCT | Recommend stopping O2 for SpO2 above 96% (strong recommendation) | Expert Guidelines |  |
| O-Driscoll BR et al. 2017^3^ | III | Practice Guidelines | |  | Recommend initial O2 titration 94-98%  COPD patients aim for SpO2 88-92% if hypercarbic 94-98% if not hypercarbic | Expert Guidelines |  |
| Bosson N et al.^4^ | III | Practice Guidelines, Case Scenarios | |  | Recommend initial O2 titration 94-98%  COPD patients aim for SpO2 88-92% |  |  |

Levels: I – strong evidence exists. II – fair evidence exists. III – weak evidence exists

**REFERENCES:**

1. Chu DK, Kim LH, Young PJ, et al. Mortality and morbidity in acutely ill adults treated with liberal versus conservative oxygen therapy (IOTA): a systematic review and meta-analysis. Lancet 2018;391:1693-705.

2. Siemieniuk RAC, Chu DK, Kim LH, et al. Oxygen therapy for acutely ill medical patients: a clinical practice guideline. BMJ 2018;363:k4169.

3. O'Driscoll BR, Howard LS, Earis J, Mak V. British Thoracic Society Guideline for oxygen use in adults in healthcare and emergency settings. BMJ Open Respir Res 2017;4:e000170.

4. Bosson N, Gausche-Hill M, Koenig W. Implementation of a titrated oxygen protocol in the out-of-hospital setting. Prehosp Disaster Med 2014;29:403-8.

Evidentiary Table: Oxygen Treatment in COPD

| **Study** | **LOE** | **Study Design** | **Methods and Outcomes** | | **Results** | **Limitations** | **Overall** |
| --- | --- | --- | --- | --- | --- | --- | --- |
| **Titrated oxygen therapy** | | | | | | | |
| Durrington HJ et al. 2005 | III | Retrospective and Pre/Post Study | | Retrospective audit of change in oxygen guidelines  Reduction of patients receiving High concentration oxygen | Giving medics venture masks decreased patient exposure to high concentration oxygen | Retrospective analysis  Not looking at patient centered outcomes | Venturi masks can decrease the exposure to high concentration oxygen when provided to medics. |
| Wijesinghe M et al. 2011 | III | Retrospective chart review | | Examined association between oxygen therapy and severity markers in patients with AECOPD  Death, Respiratory failure, | Increased oxygen flow associated with increased risk of death and poor outcome | Bias and opportunity for cofounding factors. Unequal patient distributions between high and low flow o2 | High flow O2 is linked with increased risk of death and poor outcome |
| Cameron L et al. 2012 | II | Retrospective chart review | | Examined patients with AECOPD who arrived by ambulance comparing oxygen status by ABG with outcomes of hypercapnic respiratory failure, assisted ventilation, death | Hyperoxemia associated with adverse events. Target o2 88-92% | Retrospective. Risk of bias and confounding factors | Hyperoxemia is associated with increased risk of adverse events. Goal O2 88-92% |
| Ringbaek TJ et al. 2015 | II | Cohort | | Compared oxygen administration/saturaton/abg of 405 sequential patients with outcome of respiratory acidosis, need for assisted ventilation, LOS, and in-hospital mortality | Patients with inappropriate oxygen (above 92) therapy had a high frequency of respiratory acidosis. | Not designed to clarify optimal strategy | Patients with saturation above 92% have a high rate of respiratory acidosis |
| Austin et al. 2010  ^1^ | I | Cluster randomized controlled parallel group trial | | RCT which compared high flow oxygen to oxygen titration in the prehospital setting for COPD  Intervention: nasal prongs with goal SpO2 of 88-92% with bronchodilators  Control: high flow O2 at 8-10 L/min via non-rebreather mask  Otherwise protocol was the same: salbutamol 5mg neb, ipratropium 0.5mg neb, dexamethasone 8mg IV, and (if needed) salbutamol 200-300mg IV or 500mg IM  Outcomes: mortality, rates of hypercapnia, rate of respiratory acidosis | Risk of death was lower in the titrated oxygen group compared to high flow oxygen  Oxygen titration also reduced hypercapnia and respiratory acidosis | Lower compliance in oxygen titration arm (56% did not comply with oxygen titration protocol compared to 21% in high flow) – yet still showed benefits in titration  Low rate of ABG drawn in ED. (11% compliance) | Oxygen titration in prehospital setting significantly reduces mortality, hypercapnia, and respiratory acidosis compared to high flow oxygen in exacerbations of chronic obstructive pulmonary disease |

Levels: I – strong evidence exists. II – fair evidence exists. III – weak evidence exists

**REFERENCES:**

1. Durrington HJ, Flubacher M, Ramsay CF, Howard LS, Harrison BD. Initial oxygen management in patients with an exacerbation of chronic obstructive pulmonary disease. *Qjm.* 2005;98(7):499-504.

2. Wijesinghe M, Perrin K, Healy B, et al. Pre-hospital oxygen therapy in acute exacerbations of chronic obstructive pulmonary disease. *Internal medicine journal.* 2011;41(8):618-622.

3. Cameron L, Pilcher J, Weatherall M, Beasley R, Perrin K. The risk of serious adverse outcomes associated with hypoxaemia and hyperoxaemia in acute exacerbations of COPD. *Postgrad Med J.* 2012;88(1046):684-689.

4. Ringbaek TJ, Terkelsen J, Lange P. Outcomes of acute exacerbations in COPD in relation to pre-hospital oxygen therapy. *Eur Clin Respir J.* 2015;2.

5. Austin MA, Wills KE, Blizzard L, Walters EH, Wood-Baker R. Effect of high flow oxygen on mortality in chronic obstructive pulmonary disease patients in prehospital setting: randomised controlled trial. *BMJ.* 2010;341:c5462.

Evidentiary Table: Steroids for Asthma and COPD

| **Study** | **LOE** | **Study Design** | **Methods and Outcomes** | | **Results** | **Limitations** | **Overall** |
| --- | --- | --- | --- | --- | --- | --- | --- |
| **Steroids for bronchospasm** | | | | | | | |
| Rowe BH et al. 2001^1^ | I | Systematic Review and Meta-analysis | | Selected RCTs of ED asthma patients treated with IV or IM steroids.  Examined pulmonary function and admission rates | Early administration reuced admission rates, NNT 8. Oral administration effective in children (not studied in adults) | Heterogeneity  Did not look at PO administration for adults. | Systemic steroids reduced admission rates, more marked with those with severe asthma and those not already on systemic steroids. |
| Rodrigo G et al. 1999^2^ | I | Systematic Review and Meta-analysis | | RCTs of patients with asthma exacerbations treated with parental steroids.  PFT, hospitalization | Parenteral CCS don’t improve need for hospitalization or PFTs at 3 hours. | Heterogeneity, Time until observed effect | No improvement at 3 hours. |
| Rowe BH et al. 1999^3^ | I | Systematic Review and Meta-analysis | | Selected RCTs of patients over 24 months who received IV/IM/oral glucocorticoids for asthma exacerbation  Examined hospitalization rate, relapse rate, pulmonary functions, quality of life, clinical scores | Reduction in admission rates.  Oral administration favored over IV. | Heterogeneity of data.  Age of studies | Systemic steroids reduced admission rates. Oral administration is supported. |
| Lin RY et al. 1999^4^ | I | Randomized Control Trial | | Double-blind, placebo-controlled RCT  Asthma pts with PEFRs <50% predicted after albuterol during exerabtion given either 125 mg methylprednisolone or saline  Outcome PEFR change | Significantly greater improvement of PEFR in treatment group. Also improvement in HR compared to placebo | Slightly fewer pts in status asthmaticus in treatment group | Systemic steroids led to greater improvement of pulmonary function tests |
| Knapp B et al.^5^ | II | Retrospective chart review | | Patients with moderate-severe asthma given 125mg methylprednisolone vs not | 31 patients identified given steroids by EMS, 33 given steroids in ED.    12.9% admission rate when administerd by EMS vs 33.3% | Retrospective, not randomized, variations in physician/ED care | EMS administration led to reduced admission rates |
| Walters JA et al. 2014^6^ | I | Systematic Review and Meta-analysis | | Found RCTs comparing steroids administered orally or parenterally for COPD  Outcomes included pulmonary function, relapse rate, treatment failure | Steroids had reduced treatment failure, reduced relapse rate, Improved pulmonary function.  No difference PO vs parenteral | Heterogeneity of data | Improved treatment parameters, relapse rate, pulmonary function.  No differences by route of administration |
| Lindenauer et al.^7^ | II | Retrospective cohort study | | Non-ICU COPD exacerbation patients who received low PO dose vs high IV dose  Examined treatment failure (intubation after 2^nd^ hospital day, mortality, readmission rate, length of stay) | No difference in mortality.  Risk of treatment failure lower in orally treated patients. As was length of stay and cost. | Retrospective | Among patients hospitalized for COPD exacerbations, outcomes were not worse if steroids were administered orally. |

Levels: I – strong evidence exists. II – fair evidence exists. III – weak evidence exists

**REFERENCES:**

1. Rowe BH, Spooner C, Ducharme FM, Bretzlaff JA, Bota GW. Early emergency department treatment of acute asthma with systemic corticosteroids. Cochrane Database Syst Rev 2001:CD002178.

2. Rodrigo G, Rodrigo C. Corticosteroids in the emergency department therapy of acute adult asthma: an evidence-based evaluation. Chest 1999;116:285-95.

3. Rowe BH, Keller JL, Oxman AD. Effectiveness of steroid therapy in acute exacerbations of asthma: a meta-analysis. Am J Emerg Med 1992;10:301-10.

4. Lin RY, Pesola GR, Bakalchuk L, et al. Rapid improvement of peak flow in asthmatic patients treated with parenteral methylprednisolone in the emergency department: A randomized controlled study. Ann Emerg Med 1999;33:487-94.

5. Knapp B, Wood C. The prehospital administration of intravenous methylprednisolone lowers hospital admission rates for moderate to severe asthma. Prehosp Emerg Care 2003;7:423-6.

6. Walters JA, Tan DJ, White CJ, Gibson PG, Wood-Baker R, Walters EH. Systemic corticosteroids for acute exacerbations of chronic obstructive pulmonary disease. Cochrane Database Syst Rev 2014:CD001288.

7. Lindenauer PK, Pekow PS, Lahti MC, Lee Y, Benjamin EM, Rothberg MB. Association of corticosteroid dose and route of administration with risk of treatment failure in acute exacerbation of chronic obstructive pulmonary disease. JAMA 2010;303:2359-67.

**Supplementary Table.** The protocols of the 33 Local EMS Agencies (LEMSAs) in California were examined regarding specific treatments in the care of patients with respiratory distress. There is variability among the different agency protocols. This is most pronounced in the titration of oxygen for patients with and without COPD.

| LEMSA^[[1]](#footnote-1)^ | SpO2^[[2]](#footnote-2)^ Guidelines | SpO2 Reduction for COPD^[[3]](#footnote-3)^ | Bronchospasm | | | Acute Pulmonary Edema | | NIPPV^[[4]](#footnote-4)^ for Bronchospasm | NIPPV for Pulmonary Edema | NIPPV for Undifferentiated Distress |
| --- | --- | --- | --- | --- | --- | --- | --- | --- | --- | --- |
|  |  |  | Albuterol | Ipratropium | Steroids | Nitroglycerin | Furosemide |  |  |  |
| Alameda | 94%-99% | 92-94% | Yes | Yes | No | Yes | No | Yes | Yes | Yes |
| Central California | 95-100% | No | Yes | No | No | Yes | No | Yes | Yes | No (Bronchospasm, CHF^[[5]](#footnote-5)^, Bronchospasm with anaphylaxis) |
| Coastal Valleys | 94-98% (if receiving O2) | No | Yes | Yes | No | Yes | No | Yes | Yes | Yes |
| Contra Costa | 93-95% (if receiving O2) | 88-92% | Yes | No | No | Yes | No | Yes | Yes | Yes |
| El Dorado |  | No | Yes | Yes | No | Yes | No | Yes | Yes | Yes |
| Imperial | 94-100% | No | Yes | No | No | Yes | No | Yes | Yes | Yes |
| Inland Counties |  | No | Yes | No | No | Yes | No | Yes | Yes | No (Bronchospasm, CHF, Allergy/Anaphylaxis) |
| Kern | 94-100% | No | Yes | Yes | No | Yes | No | Yes | Yes | Yes |
| Los Angeles | 94-100% | No | Yes | No | No | Yes | No | Yes | Yes | Yes |
| Marin | 94-99% | No | Yes | Yes | No | Yes | No | Yes | Yes | Yes |
| Merced | 95-100% | No | Yes | No | No | Yes | No | Yes | Yes | No (Bronchospasm, CHF, Allergy causing bronchospasm) |
| Monterey | 94-100% | 92% | Yes | No | No | Yes | No | Yes | Yes | No |
| Mountain Valley |  | No | Yes | No | No | Yes | No | Yes | Yes | Yes |
| Napa | 94-100% | No | Yes | Yes | No | Yes | No | Yes | Yes | Yes |
| Nor-Cal |  | No | Yes | Yes | No | Yes | No | Yes | Yes | Yes |
| North Coast |  | No | Yes | Yes | No | Yes | Yes (With transport over 45 min, with EMS agency approval) | Yes | Yes | Yes |
| Orange | 95-100% | No | Yes | No | No | Yes | No | Yes | Yes | Yes |
| Riverside |  | No | Yes | Yes | No | Yes | No | Yes | Yes | No (CHF, COPD, Asthma) |
| Sacramento | 94-100% | No | Yes | No | No | Yes | No | Yes | Yes | Yes |
| San Benito | 94-100% | No | Yes | Yes | No | Yes | No | Yes | Yes | Yes |
| San Diego |  | No | Yes | Yes | No | Yes | No | Yes | Yes | Yes |
| San Francisco | 94-95% (if receiving O2) | No | Yes | No | No | Yes | No | Yes | Yes | Yes |
| San Joaquin |  | No | Yes | Yes | No | Yes | No | Yes | Yes | Yes |
| San Luis Obispo | 94-100% | No | Yes | No | No | Yes | No | Yes | Yes | Yes |
| San Mateo |  | No | Yes | No | No | Yes | No | Yes | Yes | Yes |
| Santa Barbara | 94-100% | No | Yes | No | No | Yes | No | Yes | Yes | Yes |
| Santa Clara | 94-100% | No | Yes | No | No | Yes | No | Yes | Yes | Yes |
| Santa Cruz | 94-100% | No | Yes | No | No | Yes | No | Yes | Yes | Yes |
| Sierra Sacramento Valley | 94-100% | No | Yes | Yes | No | Yes | No | Yes | Yes | Yes |
| Solano |  | Consider Lower Dose | Yes | No | No | Yes | No | Yes | Yes | No (CHF, Bronchospasm, Near drowning) |
| Tuolumne |  | No | Yes | Yes | No | Yes | No | Yes | Yes | No (CHF, Near drowning, Bronchospasm, Carbon monoxide poisoning) |
| Ventura |  | No | Yes | No | No | Yes | No | Yes | Yes | Yes |
| Yolo | 94-100% | No | Yes | Yes | No | Yes | No | Yes | Yes | Yes |
| # of Agencies with Protocols | 21/33 |  |  |  |  |  |  |  | 100% |  |

1. Local EMS Agency [↑](#footnote-ref-1)
2. Oxygen Saturation [↑](#footnote-ref-2)
3. Chronic Obstructive Pulmonary Disease [↑](#footnote-ref-3)
4. Non-Invasive Positive Pressure Ventilation [↑](#footnote-ref-4)
5. Congestive Heart Failure [↑](#footnote-ref-5)
